# NF-κB p65 directs sex-specific neuroprotection in human neurons

**DOI:** 10.1038/s41598-018-34394-8

**Published:** 2018-10-30

**Authors:** Lucia M. Ruiz-Perera, Lennart Schneider, Beatrice A. Windmöller, Janine Müller, Johannes F. W. Greiner, Christian Kaltschmidt, Barbara Kaltschmidt

**Affiliations:** 1 Molecular Neurobiology, University of Bielefeld, Bielefeld, Germany; 2 Department of Cell Biology, University of Bielefeld, Bielefeld, Germany

## Abstract

Protection of neurons against oxidative stress is crucial during neuronal development, maintenance and for treating neurodegenerative diseases. However, little is known about the molecular mechanisms underlying sex-specific maturation and survival of neurons. In the present study, we demonstrate NF-κB-p65 mediated neuroprotection in human glutamatergic neurons differentiated from inferior turbinate stem cells (ITSCs) in a sex-dependent manner. We successfully differentiated ITSCs into MAP-2^+^/NF200^+^/Synaptophysin^+^/vGlut2^+^-glutamatergic neurons *in vitro* and *ex vivo* and validated their functionality. TNF-α-dependent NF-κB-p65 activation was accompanied by significant neuroprotection against oxidative stress-induced neuronal death, which was surprisingly higher in neurons from female donors. Accordingly, sex-specific neuroprotection of female neurons was followed by an increased expression of special NF-κB target genes SOD2 and IGF2. Among these, SOD2 is a well known gene protecting cells against oxidative stress resulting in longevity. In addition, IGF2 is known to promote synapse formation and spine maturation, and it has antioxidant and neuroprotective effects against oxidative damage. In conclusion, we show that NF-κB-p65 is a key player in neuroprotection of human neurons, however the protective gene expression program beneath it differs between sexes. Our findings are in accordance with the increasing evidences pointing towards sex-specific differences in risk and severity of neurodegenerative diseases.

## Introduction

Acute and chronic nervous system damage in response to an insult such as oxidative stress is directly associated to neuronal death and degeneration^1^. Thus, appropriate neuroprotection remains as a crucial parameter for effective treatment of neurodegenerative diseases. Interestingly, increasing evidences point towards sex-specific differences in risk, severity and progression of neurodegenerative diseases such as Parkinson’s (PD) or Alzheimer’s disease (AD) or in case of Ischemic stroke^2–4^. In particular, female AD patients were reported to not only have an increased risk of developing AD compared to age-matched men^5^, but also showed a significantly elevated age-related decline of cognition^3,6^. On the contrary, PD was shown to have a greater prevalence and occurred in an earlier age in men compared to woman^2^. Although neurodegenerative diseases and preventive neuroprotective mechanisms^7^ seem to be subjected to sex-dependent differences, little is known about the underlying molecular mechanisms particularly regarding maturation and survival of neurons differentiated from human stem cells.

The transcription factor NF-κB (nuclear factor kappa-light-chain-enhancer of activated B-cells) is involved in a broad range of cellular processes such as cell survival, growth, stress, immune and inflammatory responses^8^. Within the murine nervous system, the NF-κB heterodimers c-Rel/p65 and p50/p65, and p50 homodimers play an important role during development^9^, while the activity of p50/p65 was shown to be predominant in the adult brain^10^. Activation of NF-κB can be triggered by multiple stimuli such as cytokines like tumour necrosis factor-α (TNF-α) or neurotransmitters like AMPA or glutamate in mouse and rat cerebellar granule cells^11,12^. In murine neurons, NF-κB signalling and its target genes are involved in neuroprotection/degeneration^13^, neurite growth^14^, the formation of dendritic spines and their functionality^15^, axonal outgrowth^16^ and synaptic plasticity^17,18^. Activation of NF-κB in human and murine cells is also known to be caused by oxidative stress, an increase in intracellular reactive oxygen species (ROS) such as H_2_O_2_, superoxide (O_2_^−^), or hydroxyl radical (OH)^19^. Whithin the nervous system, oxidative stress leads to activation of NF-κB with a direct linkage to several neurological diseases and brain damage^20^. In functional neurons from humans or mice, activation of various glutamate receptors also induces oxidative stress^21^. On the contrary, reactive oxygen intermediate hydrogen peroxide (H_2_O_2_) is known to act as a second messenger despite its cytotoxicity^20,22^. In primary rat cerebellar granule cells, the direct exogenous addition of H_2_O_2_ to culture medium activates NF-κB^23^, as well as it was observed previously in different human cell lines^22,24,25^. In human and mouse embryonic stem cells, metabolic oxidation is known to directly regulate cell differentiation^26^. Maintenance of redox balance was further shown to be crucial for stemness and self-renewal of hematopoietic stem cells (HSCs) and neural stem cells (NSCs)^27^ from mice and humans. On the other hand, NF-κB signalling is directly linked to proliferation of rat NSCs^28^ and early neuronal differentiation of mouse NSCs^29^, although its direct role in protection of human stem cell-derived neurons against oxidative stress still remains unclear.

In the present study, we demonstrate a neuroprotective role of NF-κB-p65 through maturation of human glutamatergic neurons derived from neural crest-derived stem cells (NCSCs) after oxidative stress insult. During vertebrate development, neural crest cells migrate from the border between neural plate and non-neural ectoderm and give rise to a wide variety of cell types like neurons, glial cells, or melanocytes^30^. Pursuing their role in development, neural crest cells also persist into adulthood as NCSCs within various tissues, including skin^31^, cornea^32^, periodontal ligament^33^, palate^34^ and pulp of teeth^35^. A particularly interesting population of NCSCs has been found within the respiratory epithelium in the inferior turbinate of the human nose. Inferior turbinate stem cells (ITSCs) are able to differentiate into a wide variety of cell types from mesodermal and neuro-ectodermal lineages, such as chondrocytes, osteocytes, adipocytes, and glutamatergic as well as dopaminergic neurons^36–38^. Due to their capability to efficiently give rise to neuronal cell types, ITSCs harbor great potential for the treatment of neurodegenerative diseases^38^. Thus, ITSC-derived neurons served as an ideal model system for determining molecular mechanisms regulating maturation and survival of human neurons in the present study. We differentiated ITSCs into MAP2^+^/NF200^+^/Synaptophysin^+^/vGlut2^+^-glutamatergic neurons, which we successfully stimulated with AMPA or glutamate leading to activation of NF-κB-p65. TNF-α-dependent stimulation of NF-κB-p65 in ITSC-derived neurons resulted in a significant neuroprotective effect against oxidative stress-induced cell death. ITSC-derived neurons from female donors further showed a significantly elevated sensitivity to H_2_O_2_ as well as a 2-fold increase in TNF-α-dependent neuroprotection compared to neurons from male donors. Our findings reveal NF-κB-p65 as a key player in neuroprotection of NCSC-derived neurons in a sex-dependent manner, indicating the pivotal role of NF-κB-signalling during stem cell-based neuronal differentiation.

## Results

### Inferior turbinate stem cells efficiently differentiate into glutamatergic neurons *in vitro*

**Figure 1:**
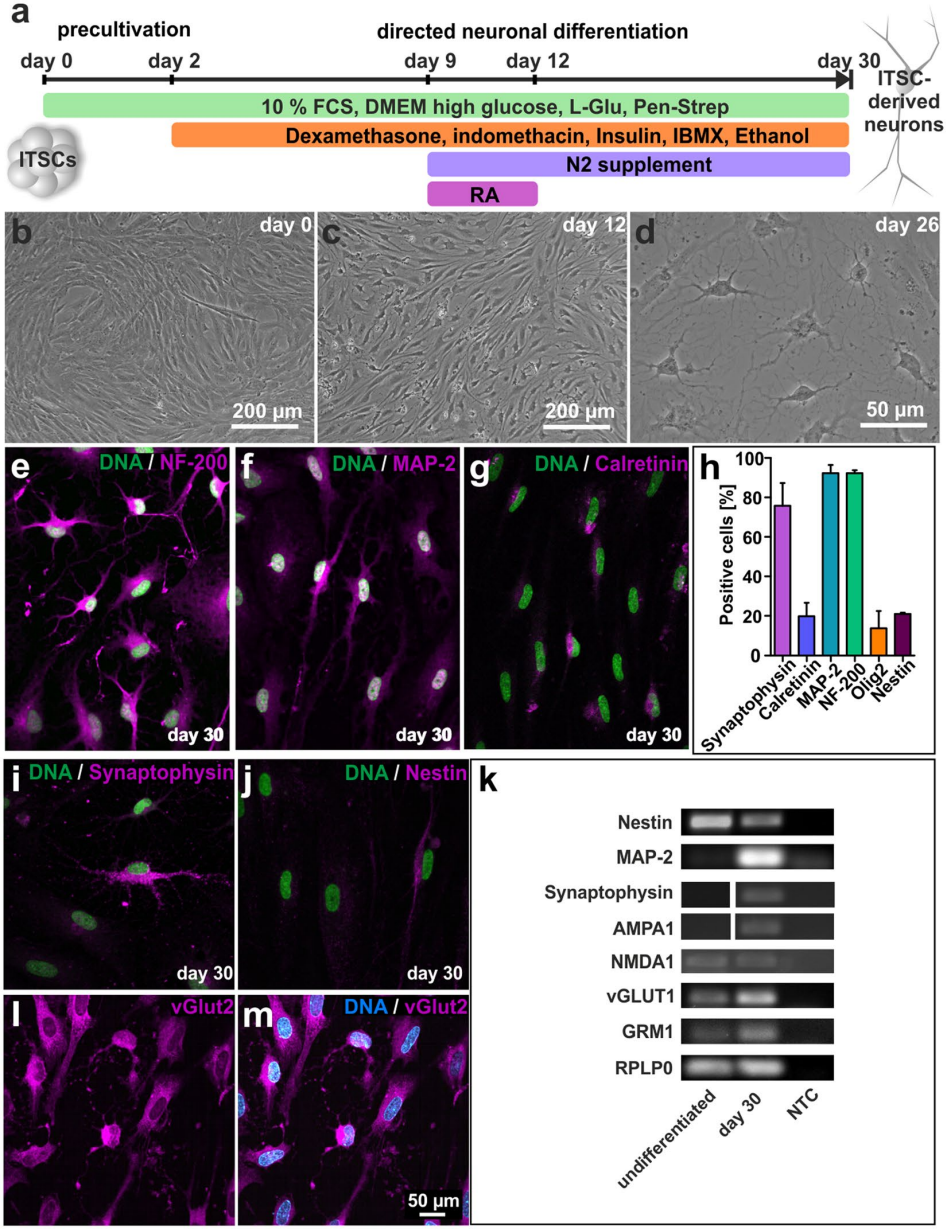
Adult human neural crest-derived stem cells from the inferior turbinate (ITSCs) are able to efficiently differentiate into glutamatergic neurons. (**a**) Schematic view of the neuronal differentiation procedure. (**b**–**d**) ITSCs changed their morphology towards a neuronal phenotype during directed neuronal differentiation. (**e**–**g**) Immunocytochemistry of ITSCs after 30 days of differentiation depicting the presence of positive cells for NF200, MAP-2 and calretinin (perinuclear region). (**h**) Quantification of immunocytochemical analyses showing the percentage of Synaptophysin^+^ (75,77% ± 11,55%), Calretinin^+^ (19,77% ± 6,85%), MAP-2^+^ (92,28% ± 4,20%), NF-200^+^ (92,28% ± 1,45%), Olig2^+^ (13,70% ± 8,74%) and Nestin^+^ (13,70% ± 0,63%) ITSC-derived neurons after 30 days of neuronal differentiation (Mean ± SEM; n = 3). (**I**,**j**) Differentiated ITSCs were positive for Synaptophysin, while small population of cells remained Nestin-positive. (**k**) RT-PCR of differentiated ITSCs showing the down-regulation of Nestin and the up-regulation of MAP-2, Synaptophysin, AMPA receptor subunit 1, NMDA Receptor subunit 1, vesicular glutamate transporter 1, and glutamate metabotropic receptor 1 after neuronal differentiation at the mRNA level. RPLP0 served as housekeeping gene. NTC: non-template-control. The grouped gels were cropped from different parts of the same gel or from different gels, full-length gels are shown in the Supplementary Fig. S1. (**l**,**m**) Most ITSC-derived neurons were vGlut2^+^.

In order to gain an appropriate model system for studying the role of NF-κB in neuroprotection during maturation of human NCSC-derived neurons, ITSCs were cultivated following a directed neuronal differentiation procedure for 30 days (Fig. 1a)^36,38^. Exposure of ITSCs to a neuronal induction medium for 28 days resulted in a neuronal-like morphology indicated by retraction of the cytoplasm towards the nucleus, and extended cellular processes resulting in neurite outgrowth (Fig. 1b–d). Immunocytochemical analyses confirmed the presence of the mature neuronal markers Neurofilament 200 (Fig. 1e,h; 92,28% ± 1,45%), MAP-2 (92,28% ± 4,20%; Fig. 1f,h) and Synaptophysin (75,77% ± 11,55%; Fig. 1i,h). Interestingly, 19,77% ± 6,85% of ITSC-derived neurons were positive for Calretinin (Fig. 1g,h), while a small subpopulation of 13,70% ± 8,74% differentiated ITSCs expressed the glial marker Olig-2 (Fig. 1h). Further validating their successful differentiation, only 20,96% ± 0,63% of ITSCs showed co-expression of Nestin (Fig. 1j) after directed differentiation. Accordingly, RT-PCR analyses depicted a decrease in expression of Nestin as well as an increased expression of MAP-2 and Synaptophysin in ITSC-derived neurons (Fig. 1i). Characterizing these neurons in more detail, we observed AMPA receptor subunit 1, NMDA receptor subunit 1, glutamate metabotropic receptor 1 (GRM1) as well as the vesicular glutamate transporter 1 (VGLUT1) were robustly expressed (Fig. 1k). Immunocytochemistry further confirmed the glutamatergic phenotype of ITSC-derived neurons by revealing vGlut2-expression on the protein level (Fig. 1l,m).

### Integration and differentiation of ITSC-derived glutamatergic neurons after transplantation into *ex vivo*-cultivated murine organotypic hippocampal slices

**Figure 2:**
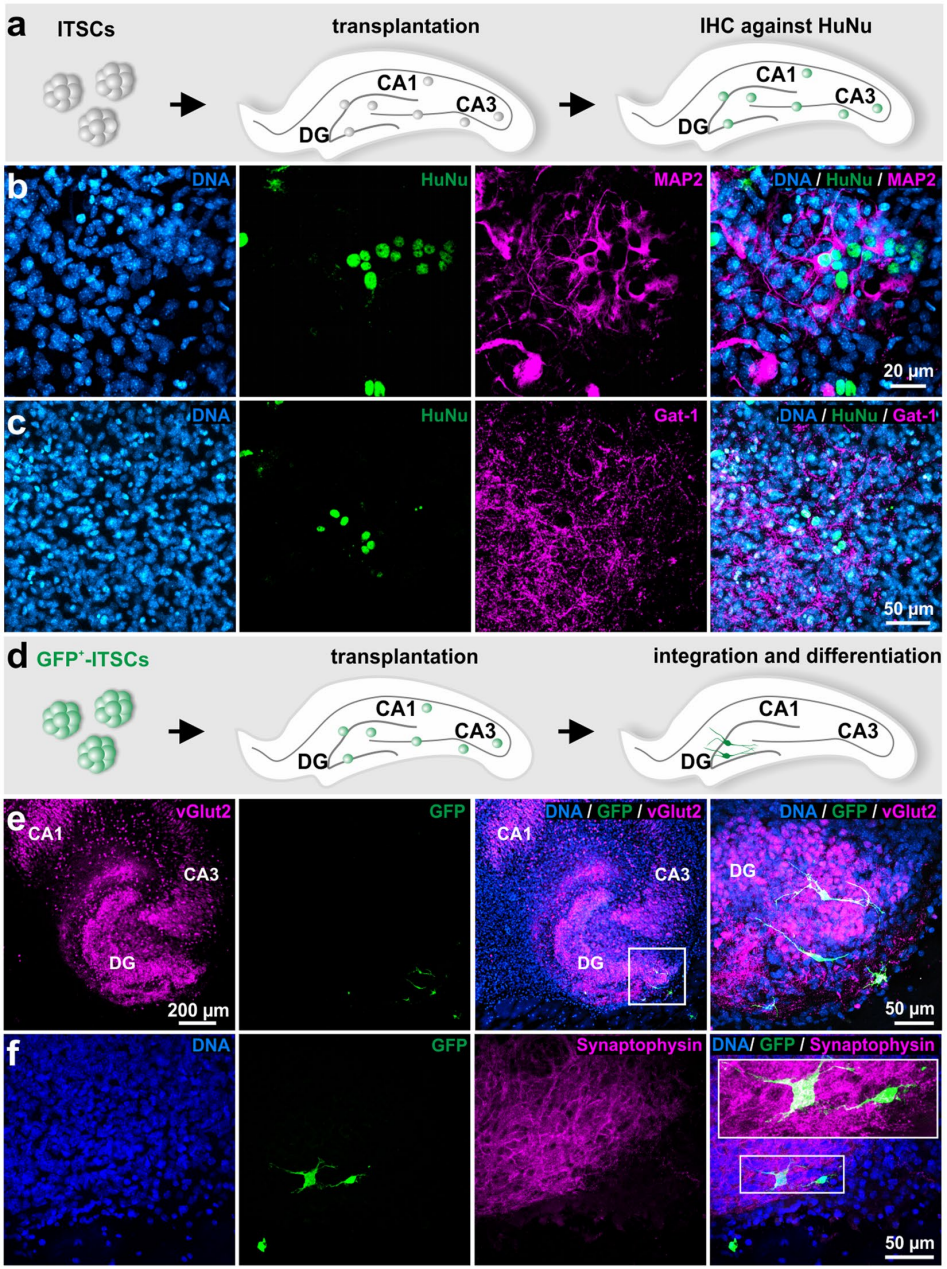
ITSCs differentiate into glutamatergic neurons after transplantation into *ex vivo*-cultivated murine organotypic hippocampal slices. (**a**) Schematic view showing experimental design of ITSC-transplantation into organotypic hippocampal slices. (**b**) Immunocytochemistry showing differentiation of ITSCs into MAP-2 and Gat-1 positive neurons, indicating successful integration of transplanted ITSCs into organotypic hippocampal slices. (**d**) Schematic view showing the transplantation procedure of GFP-positive ITSCs into organotypic hippocampal slices. E: GFP^+^-ITSCs integrated within the dentate gyrus of the hippocampus and exhibited a neuronal phenotype accompanied by expression of vGlut2 and Synaptophysin.

In addition to their efficient neuronal differentiation *in vitro*, we evaluated the ability of ITSCs to integrate and differentiate within a neural environment by transplanting undifferentiated stem cells into murine organotypic hippocampal slices (Fig. 2a). Transplanted human ITSCs were able to integrate in the murine neural tissue and differentiated into MAP-2^+^ and Gat-1^+^ neurons after 14 days of co-cultivation (Fig. 2b,c). Furthermore, GFP-positive ITSCs integrated particularly into the dentate gyrus of organotypic hippocampal slices, where they exhibited a clear neuronal phenotype accompanied by expression of vGlut2 and Synaptophysin on protein level (Fig. 2d–f). These findings confirmed that ITSCs are also able to give rise to excitatory glutamatergic neurons within the proper neural environment.

### ITSC-derived glutamatergic neurons show AMPA or glutamate-dependent activation of NF-κB-p65

**Figure 3:**
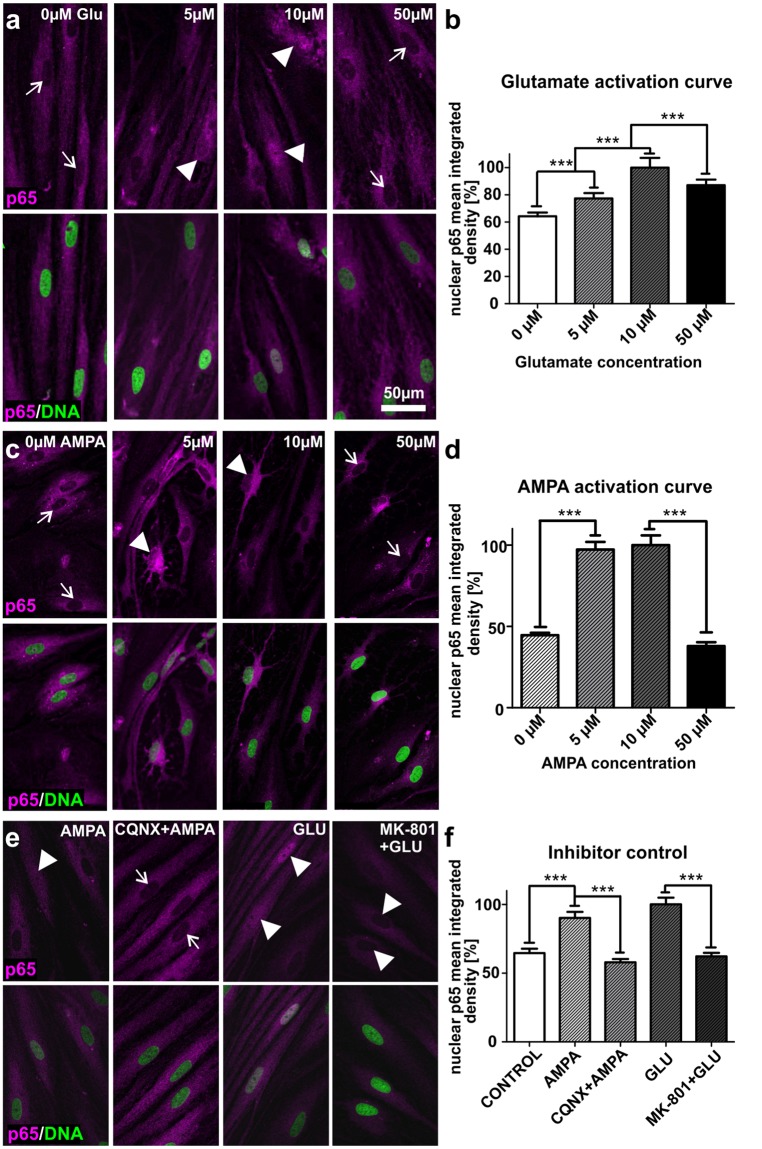
Stimulation of glutamatergic neurons derived from ITSCs leads to significantly increased nuclear translocation of NF-κB-p65. (**a**,**b**) Immunocytochemistry and respective quantification of nuclear mean integrated density of NF-κB-p65 revealed significantly increased nuclear translocation of NF-κB-p65 in ITSC-derived neurons after glutamate (GLU)-dependent stimulation (arrowheads) compared to control (arrows). Mean values were normalized to the highest value. (**c**,**d**) AMPA-dependent stimulation resulted in significantly increased nuclear translocation of NF-κB-p65 in ITSC-derived neurons (arrowheads) compared to control (arrows). Mean values were normalized to the highest value. (**e**,**f**) Pre-treatment of ITSC-derived neurons with dibenzocyclohepteneimine (MK-801) or 6-cyano-7-nitroquinoxaline-2, 3-dione (CQNX) prior to GLU or AMPA-treatment led a significant decrease in nuclear translocation of NF-κB-p65 (arrows) compared to GLU or AMPA-dependent stimulation (arrowheads). Mean values were normalized to the highest value. Statistical analysis was performed using Graph Pad Prism 5. Normality of the data sets was refuted after analysis using Kolmogorov-Smirnov and Shapiro-Wilk normality tests. Non-parametric Mann-Whitney test was further used (***p ≤ 0.001), error bars indicate the standard error of the mean (SEM).

We next investigated the capability of ITSC-derived neurons to respond to the excitatory neurotransmitter glutamate (GLU) or its agonist α-amino-3-hydroxyl-5-methyl-4-isoxazole-propionate (AMPA). Stimulation with GLU or AMPA resulted in a significant increase in nuclear translocation of NF-κB-p65 in a dose-dependent manner (5–10 µM) in comparison to untreated neurons. On the contrary, treatment with 50 µM GLU or AMPA led to a significant decline in NF-κB-p65 nuclear translocation compared to 10 µM-treatment (Fig. 3a–d). We also observed high levels of basal NF-κB-activity (Fig. 3a–d), in accordance to the already described constitutive activation of NF-κB particularly in glutamatergic neurons^39^. Treatment of ITSC-derived neurons with their respective inhibitors 6-cyano-7-nitroquinoxaline-2, 3-dione (CQNX) or dibenzocyclohepteneimine (MK-801) prior to application of GLU (10 µM) or AMPA (10 µM) resulted in a significantly reduced translocation of NF-κB-p65 into the nucleus compared to the stimulation approaches (Fig. 3e,f). These findings provide pharmacological evidence that both kinds of receptors were expressed in human ITSC-derived glutamatergic neurons, which in turn were observed to be fully functional after 30 days of differentiation.

### Stimulation with TNF-α leads to significantly increased nuclear translocation of NF-κB-p65 in ITSC-derived glutamatergic neurons

**Figure 4:**
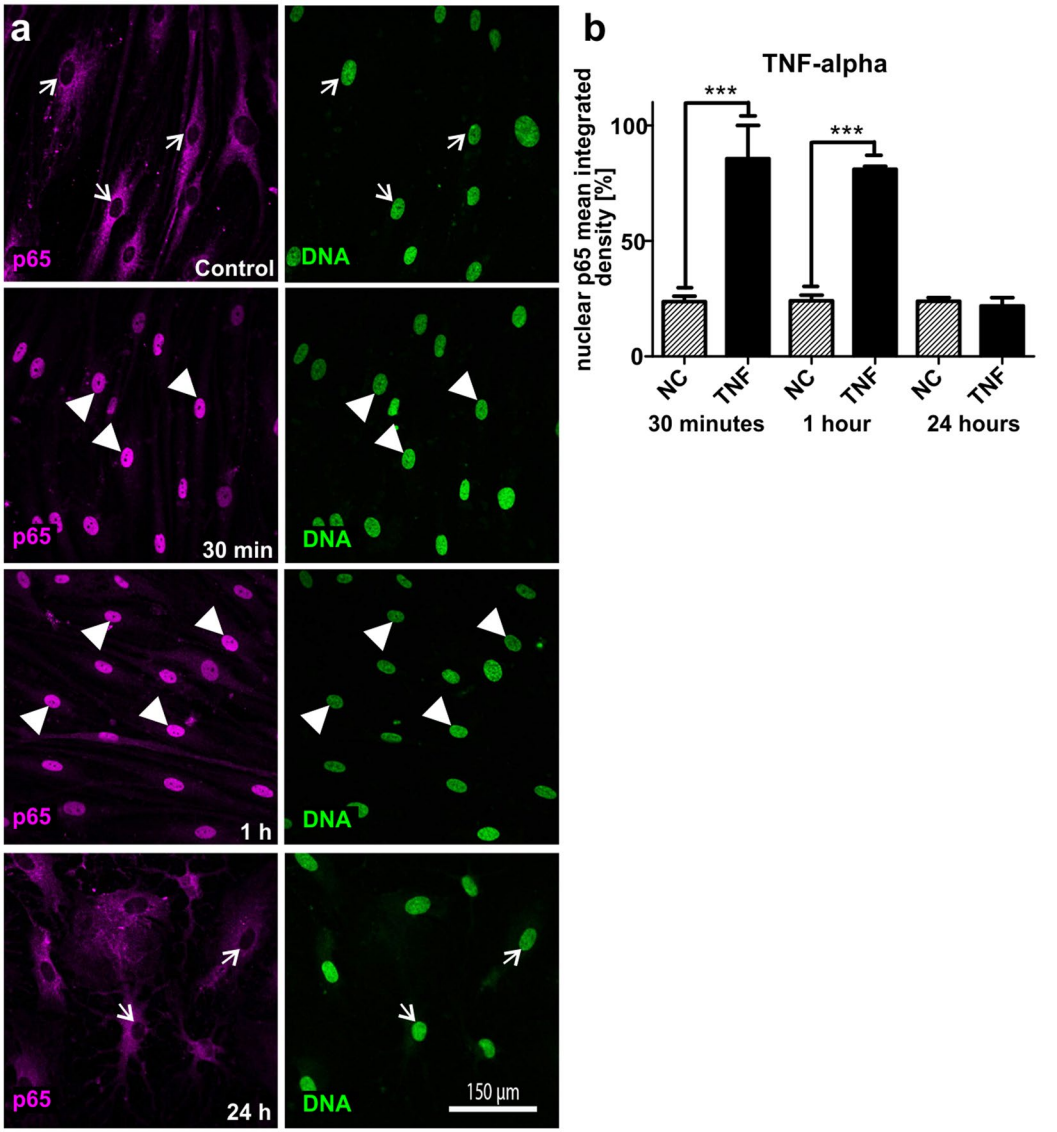
TNF-α-dependent stimulation of glutamatergic neurons derived from ITSCs leads to nuclear transloaction of NF-κB-p65. (**a**) Immunocytochemistry of ITSC-derived neurons (30 days of differentiation) showed nuclear translocation of NF-κB-p65 after treatment with TNF-α for 30 min or 1 h (arrowheads), whereas control and 24 h-TNF-α-treatment did not result in nuclear translocation of NF-κB-p65 (arrows). (**b**) Quantification of nuclear mean integrated density of NF-κB-p65 confirmed the significant increase in nuclear NF-κB-p65 after TNF-α-treatment of ITSC-derived neurons for 30 min or 1 h compared to 24 h and untreated control. Mean values were normalized to the highest value. Statistical analysis was performed using Graph Pad Prism 5. Normality was refuted after analysis using Kolmogorov-Smirnov and Shapiro-Wilk normality tests. Non-parametric Mann-Whitney test was further used. ***p ≤ 0.001, error bars indicate the standard error of the mean (SEM).

After validating human ITSC-derived neurons as a model system for studying the role of NF-κB during maturation, we investigated the potential of TNF-α to stimulate NF-κB in these neurons. Stimulation of ITSC-derived neurons with TNF-α for 30 minutes or 1 hour resulted in nuclear translocation of NF-κB-p65 (Fig. 4a, arrowheads) in comparison to untreated neurons or 24 h of TNF-α-treatment (Fig. 4a, arrows). Quantification of the NF-κB-p65 nuclear mean integrated density clearly validated these dynamics by showing a highly significant increase in nuclear NF-κB-p65 fluorescence after 30 minutes (93,96% ± 6,04%) and 1 hour (88,00% ± 12,00%) of TNF-α-treatment compared to untreated controls (<20%) (Fig. 4b). Accordingly, stimulation of ITSC-derived neurons with TNF-α for 24 hours did not result in a significantly different nuclear NF-κB-p65 fluorescence intensity compared to control (Fig. 4b).

### TNF-α-pre-treatment of human ITSC-derived glutamatergic neurons leads to increased NF-κB-p65-activity upon oxidative stress insult

We further analyzed the effect of H_2_O_2_-mediated oxidative stress insult on the activity of NF-κB-p65 in ITSC-derived neurons. Application of H_2_O_2_ for 25 h on human glutamatergic neurons differentiated for 30 days led to significantly increased nuclear translocation of NF-κB-p65 in comparison to control. In order to analyze a potential neuroprotective role of NF-κB, we performed a pre-treatment using 10 ng/ml TNF-α during 2 hours prior to oxidative stress insult.

**Figure 5:**
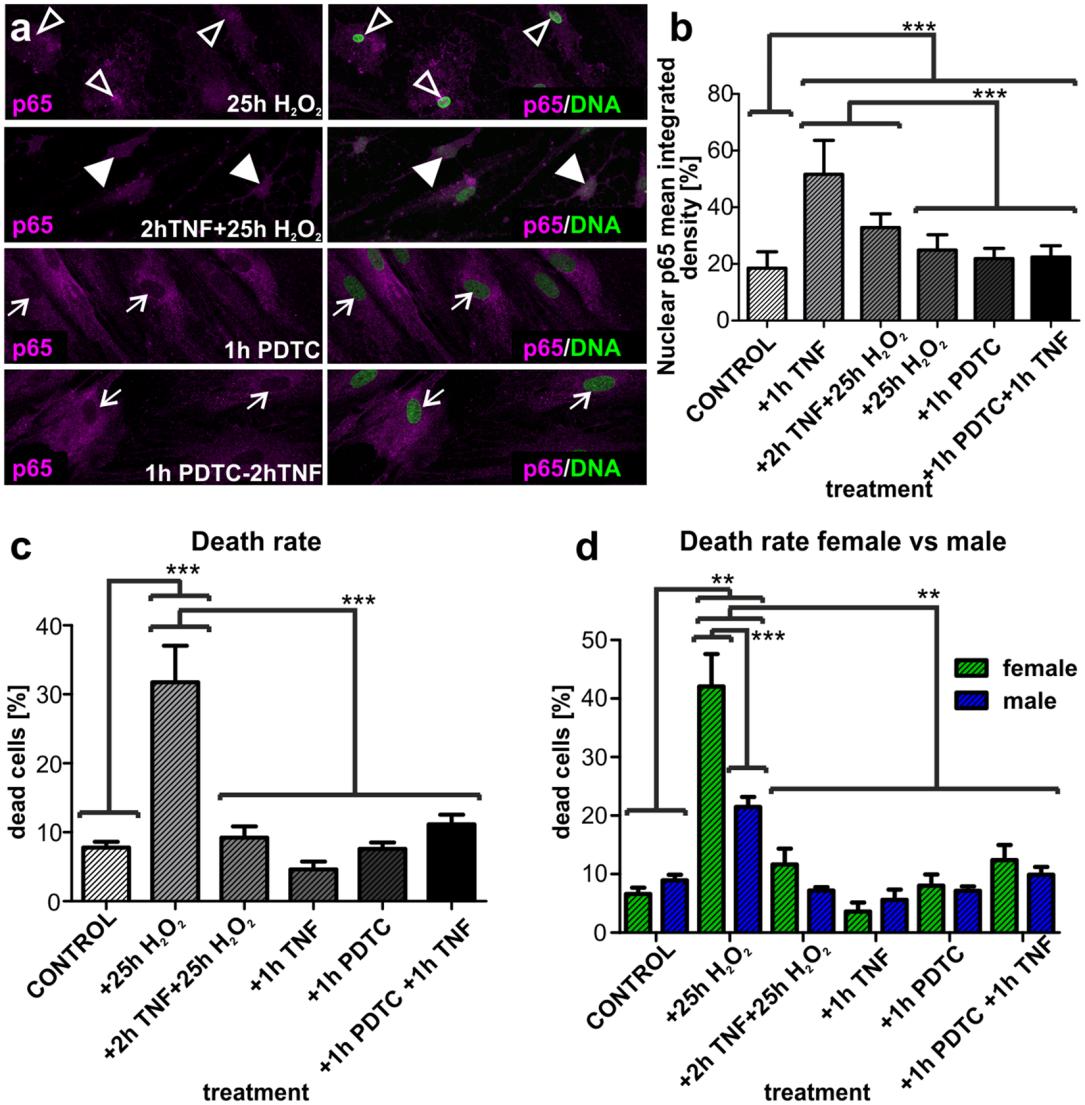
Treatment of ITSC-derived glutamatergic neurons with TNF-α prevents from oxidative stress-mediated cell death in a sex-dependent manner. (**a**) Immunocytochemistry of ITSC-derived neurons after 30 days of differentiation, after treatment with H_2_O_2_ alone, TNF-α-pre-treatment prior to H_2_O_2_, PDTC alone and PDTC followed by TNF-α against NF-κB-p65. (**b**) Quantification of immunocytochemical assays showed significantly increased nuclear translocation of NF-κB-p65 after treatment with TNF-α alone and TNF-α prior to H_2_O_2_ compared to H_2_O_2_ alone and untreated control. Pre-treatment of ITSC-derived neurons with PDTC for one hour followed by TNF-α-treatment did not result in significantly different amounts of nuclear NF-κB-p65 compared to PDTC alone. Mean values were normalized to the highest value. (**c**) Quantification of neuronal cell death showed significant death after oxidative stress insult (H_2_O_2_) compared to TNF-α/ H_2_O_2_, TNF-α, PDTC, PDTC/TNF-α and untreated control (n = 6). (**d**) Quantification of neuronal cell death after oxidative stress (H_2_O_2_), TNF-α-pre-treatment, TNF-α, PDTC, PDTC/ TNF-α and untreated control comparing sex differences (n = 3 males, n = 3 females). Data were showed not to be normally distributed using Kolmogorov-Smirnov and Shapiro-Wilk normality tests. Non-parametric Kruskal-Wallis test was further used (p ≤ 0.001), and Tukey’s post-hoc test (**p ≤ 0.01, ***p ≤ 0.001). Mean ± SEM (standard error of the mean).

Notably, TNF-α-pre-treatment of ITSC-derived glutamatergic neurons followed by H_2_O_2_-mediated oxidative stress resulted in a significant increase in nuclear translocation of NF-κB-p65 compared to the H_2_O_2_ alone or control (Fig. 5a,b, arrowheads). We further applied pyrrolidine dithiocarbamate (PDTC) as a control for guided inhibition of NF-κB. Pre-treatment of the cultivated neurons with PDTC for 1 hour followed by application of TNF-α or sole PDTC-treatment did not result in changes of nuclear translocation of NF-κB-p65 (Fig. 5a,b, arrows). Quantification of the nuclear mean integrated density for p65 indicated a small but significant increase in nuclear NF-κB-p65 in both treatments compared to the untreated negative control (Fig. 5b).

### ITSC-derived glutamatergic neurons are protected from oxidative stress-mediated cell death via TNF-α-dependent activation of NF-κB-p65

Determining the physiological consequences of TNF-α-dependent activation of NF-κB-p65 during oxidative stress, we analysed the death rate of ITSC-derived neurons after treatment with H_2_O_2_ or H_2_O_2_ after TNF-α-pre-treatment. H_2_O_2_-mediated oxidative stress led to robust and significant apoptosis of glutamatergic neurons compared to untreated control (Fig. 5c). Notably, this H_2_O_2_-mediated increase in apoptosis was significantly reduced down to a level similar to control upon TNF-α-pre-treatment prior to the oxidative stress insult (Fig. 5c). Application of TNF-α alone did not affect the survival of ITSC-derived neurons nor did the PDTC treatment, or the PDTC treatment followed by TNF-α in comparison to control (Fig. 5c).

### Sensitivity of glutamatergic neurons to ROS-mediated cell death and neuroprotection via NF-κB-p65 is dependent on the sex of the ITSC-donor

Investigating the effects of TNF-α-treatment on H_2_O_2_-mediated death of ITSC-derived neurons in more detail, we analysed the amount of apoptotic cells after oxidative stress and TNF-α-dependent neuroprotection in dependence to the sex of the ITSC-donor. We observed a significant increase in cell death of neurons differentiated from female ITSC-donors compared to their male counterparts, indicating an elevated sensitivity of human female glutamatergic neurons to oxidative stress (Fig. 5d). Pre-treatment of ITSC-derived neurons from female donors with TNF-α led to a significant and complete neuroprotection against H_2_O_2_-mediated cell death. Although neurons from male ITSC-donors were likewise protected against cell death via exposure to TNF-α, we observed a 2-fold increase in TNF-α-dependent neuroprotection in female ITSC-derived neurons compared to those differentiated from male ITSCs. These findings not only demonstrate a NF-κB-dependent neuroprotection of ITSC-derived neurons against oxidative stress-mediated cell death, but emphazise the dependence on its sensitivity to the sex of the ITSC-donor.

### TNF-α-mediated neuroprotection of ITSC-derived neurons is accompanied by sex-specific expression of NF-κB target genes

**Figure 6:**
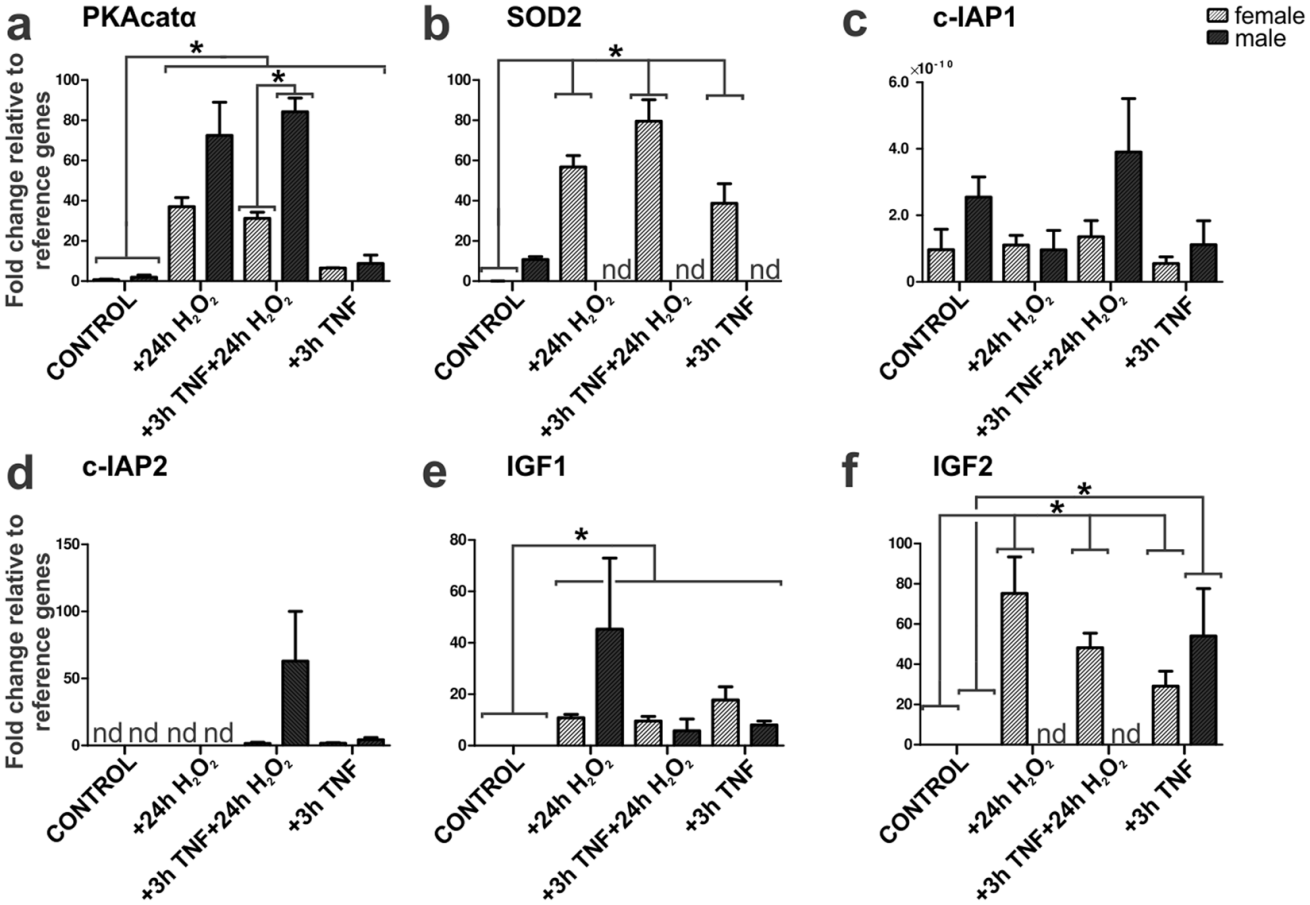
Quantitative polymerase chain reaction revealed sex-specific NF-κB-p65 target gene expression after TNF-α-dependent neuroprotection upon oxidative stress-insult. (**a**) qPCR analysis revealed increased PKAcatα mRNA levels after H_2_O_2_ alone, TNF-α-pre-treatment prior to H_2_O_2_ and TNF-α alone compared to untreated control, with a stronger effect for TNF-α/H_2_O_2_ in male compared to female ITSC-derived neurons. (**b**) SOD2 mRNA levels significantly increased in female ITSC-derived neurons compared to control and compared to their male counterparts. (**c**) qPCR analysis showing c-IAP1 mRNA levels in male and female ITSC-derived neurons. (**d**) qPCR analysis showing c-IAP2 mRNA levels in male and female ITSC-derived neurons. (**e**) qPCR analysis showing significant increased IGF1 mRNA levels compared to control in male and female ITSC-derived neurons. (**f**) IGF2 mRNA levels were significantly elevated only in female-derived neurons compared to control. Non-parametric Mann Whitney test (*p ≤ 0.05), mean ± SEM (standard error of the mean, n = 3 males, n = 3 females). Nd: Not detectable. Ct values were normalized to reference genes β-actin and RPLP0 (Ribosomal Protein Lateral Stalk Subunit P0).

To investigate the role of NF-κB-p65 in protection of ITSC-derived neurons from H_2_O_2_-mediated death in more detail, expression of NF-κB target genes was assessed by qPCR. Treatment of ITSC-derived neurons with H_2_O_2_ or TNF-α followed by H_2_O_2_ led to significantly increased expression levels of cAMP-dependent protein kinase catalytic subunit alpha (PKAcatα) compared to control. We further observed a significant increase in PKAcatα expression levels in male ITSCs-derived neurons compared to those differentiated from female ITSCs after TNF-α/H_2_O_2_-treatment (Fig. 6a). On the contrary, we observed a significant increase in manganese superoxide dismutase (Mn-SOD, SOD2) mRNA levels only in female ITSCs-derived neurons upon exposure to TNF-α, H_2_O_2_ and TNF-α/H_2_O_2_ compared to control (Fig. 6b). Expression levels of cellular inhibitor of apoptosis protein-1 and 2 (c-IAP1 and c-IAP2) showed the tendency to be elevated in male ITSCs-derived neurons after TNF-α/H_2_O_2_-treatment compared to their female counterparts (Fig. 6c,d) however no significant alteration was detectable. Treatment with TNF-α, H_2_O_2_ and TNF-α/H_2_O_2_ further resulted in significantly increased expression levels of insulin-like growth factor 1 (IGF1) in ITSCs-derived neurons compared to control (Fig. 6e), although no significant sex-dependent differences were observable. Female ITSCs-derived neurons showed significantly increased expression levels of IGF2 after H_2_O_2_ and TNF-α/H_2_O_2_-treatment compared to control (Fig. 6f), while no expression was detectable in male counterparts. However, sole treatment with TNF-α resulted in significantly increased expression levels of IGF2 in neurons from male and female donors compared to control (Fig. 6F). These findings strongly suggest a sex-specific NF-κB-p65 target gene expression in dependence to TNF-α-mediated neuroprotection during oxidative stress.

## Discussion

The present study describes for the first time a neuroprotective role of NF-κB-p65 in human ITSC-derived glutamatergic neurons after oxidative stress insult in a sex-specific manner. We successfully differentiated human neural crest-derived inferior turbinate stem cells into MAP-2^+^/NF200^+^/Synaptophysin^+^/vGlut2^+^-glutamatergic neurons by application of a directed differentiation assay or via transplantation into organotypic mouse hippocampal slice cultures. Extending our previous findings depicting vesicle recycling and calcium spiking of ITSC-derived neurons^38^, we validated their functionality by showing increased NF-κB-activity upon stimulation with the excitatory neurotransmitter glutamate or its agonist AMPA. Inhibitor controls using CQNX and MK-801 led to a decrease in glutamate or AMPA-dependent stimulation of NF-κB-activity, further validating the specificity of the respective receptors. In accordance to our findings, stimulation of ionotropic glutamate receptors was shown to activate NF-κB in primary rat cerebellar granule neurons^11,12^. Given the pivotal role of NF-κB signalling in key elements for neuronal morphology like neurite growth^14^, dendritic spine formation^15^, axonal outgrowth^16^ and synaptic plasticity^17,18^, our data suggest the participation of NF-κB in the normal physiology of the human nervous system.

In addition to its AMPA- and glutamate-dependent stimulation, we also observed a significant increase in NF-κB-activity in ITSC-derived neurons after treatment with TNF-α. In canonical NF-κB-signalling, recognition of stimuli like cytokines or neurotransmitters leads to phosphorylation of IκB kinases^40,41^, in turn resulting in phosphorylation, polyubiquitination and 26S-proteasome-mediated degradation of IκBs. Demasking of the nuclear translocation signal region of p50/p65 by degradation of IκBs is subsequently followed by translocation of p50/p65 into the nucleus and activation of target gene expression by binding to κB elements^18,42–45^. TNF-α is one of the best characterized cytokines inducing this pathway, and its receptors TNFR1 and TNFR2 are widely expressed in the nervous system both in neurons and glia^46–48^. Besides its modulatory effects of neuronal responses to excitotoxic and hypoxic insults in the nervous system^49^, the absence of TNFR was shown to result in an increased neuronal damage following either ischemic or kainic acid induced excitotoxic damage^50^. In mouse NSCs, TNF-α-mediated NF-κB signalling was reported to be required for initial neuronal differentiation^29^. In accordance, preliminary data from our lab suggests that NF-κB-c-Rel might be the relevant subunit for glutamatergic differentiation and not NF-κB-p65, having no significant differences with respect to sex (unpublished data). Extending these findings, mature human ITSC-derived glutamatergic neurons revealed a significantly increased nuclear translocation of NF-κB-p65 after TNF-α-stimulation in the present study, indicating the crucial role of NF-κB-signalling during stem cell-based neuronal differentiation and neuroprotection in humans.

Being a major cause of several neurologic diseases and brain damage^20^, oxidative stress is known to be directly caused by Alzheimer’s disease via amyloid beta peptide-dependent production of hydrogen peroxide through metal ion reduction^51,52^. In Parkinson’s disease, free radicals accumulate in the *substantia nigra pars compacta*, resulting in the formation of 6-hydroxydopamine, in turn leading to the generation of superoxide^53,54^. In the present study, H_2_O_2_-mediated oxidative stress led to cell death of human ITSC-derived glutamatergic neurons. Although NF-κB is known to be activated by oxidative stress in the nervous system^20^, several studies indicated its neuroprotective role in murine cells. Here, Heck and colleagues demonstrated an Insulin-like growth factor-1-mediated neuroprotection of rat primary cerebellar neurons against oxidative stress directly associated to activation of NF-κB^55^. Erythropoietin-mediated neuroprotection of rat cerebral cortical cell cultures from oxidative stress was also shown to occur in an NF-κB-dependent manner^56^. On the contrary, Zou and colleagues demonstrated TNF-α-treatment of rat hippocampal-entorhinal cortex slice cultures to result in increased neurotoxicity to both glutamate and oxidative stress^57^. In the present study, TNF-α-pre-treatment led to a significant decrease in H_2_O_2_-mediated cell death of ITSC-derived human neurons accompanied by a significantly increased nuclear translocation of NF-κB-p65. Our data therefore demonstrate a key role of NF-κB-p65 in protection of human stem cell-derived neurons from oxidative stress, further emphasizing the importance of NF-κB-signalling in neuroprotection^20,55,56^.

Interestingly, we further observed a significantly elevated sensitivity of ITSC-derived neurons from female donors to oxidative stress-induced cell death and to NF-κB-dependent neuroprotection compared to neurons from male donors. These findings were confirmed by a differential expression of NF-κB target genes in dependence to the sex of the ITSC-donor. Here, increased SOD2 mRNA levels observed in female but not in male ITSC-derived neurons indicated a NF-κB-associated induction of SOD2 protecting against oxidative stress-induced neuronal apoptosis. Accordingly, SOD2 expression was described to be inducible by TNF-α, having an anti-apoptotic role by directly reducing cellular ROS levels^58^. Next to SOD2, expression levels of IGF2 were significantly elevated only in female neurons after TNF-α/H_2_O_2_-treatment compared to control. IGF2 is known to promote synapse formation and spine maturation in the mouse brain^59^. Within a mouse model of Alzheimer’s disease, IGF2 administration rescued spine formation and synaptic transmission in the hippocampus^60^. In accordance to the present findings, IGF2 was reported to have an antioxidant and neuroprotective effect on oxidative damage and mitochondrial function in cultured adult rat cortical neurons^61,62^. In contrast to their female counterparts, male ITSC-derived neurons showed a significant increase in the expression level of PKAcatα after TNF-α/H_2_O_2_-treatment. Interestingly, we observed no significantly altered expression levels of the antiapoptotic proteins c-IAP1 and c-IAP2, known mediators of TNF-α-dependent neuroprotection^63^. With PKAcatα being an essential regulator in learning and memory by transducing synaptic responses through CREB signalling^64,65^ and controlling synaptic incorporation of AMPA receptors^66^, PKA-activity may directly contribute to the neuroprotective effects observed here. Although being a matter of debate, sex-dependencies in stem cell biology have already been shown in terms of autosomal gene expression^67^ and proliferation^68^, particularly regarding mouse NSCs^69,70^. Compared to their male counterparts, female muscle-derived stem cells were reported to have higher muscle regeneration efficiency in mice^71^. In terms of neuroinflammation and neuroprotection, sex-dependent differences between patients have been likewise reported in ischemic stroke^4^, PD^2^, or AD^3^. While female AD patients were described to have an increased risk of developing AD^3,6^, PD was shown to have a greater prevalence in male patients^2^. These data are in agreement with sex-specific differences found in adult murine microglia, where female microglia exhibited a neuroprotective phenotype upon ischemic insult, which was retained after being transferred into male brains^72^. In addition sex-specific differences in the expression of iNOS and NF-κB were previously reported in human polymorphonuclear neutrophils, being higher in female than in male cells^73^. In line with these findings, our data indicate for the first time a direct sex-dependent difference in neuroprotection of human stem cell-derived neurons against oxidative stress mediated by NF-κB-signalling.

**Figure 7:**
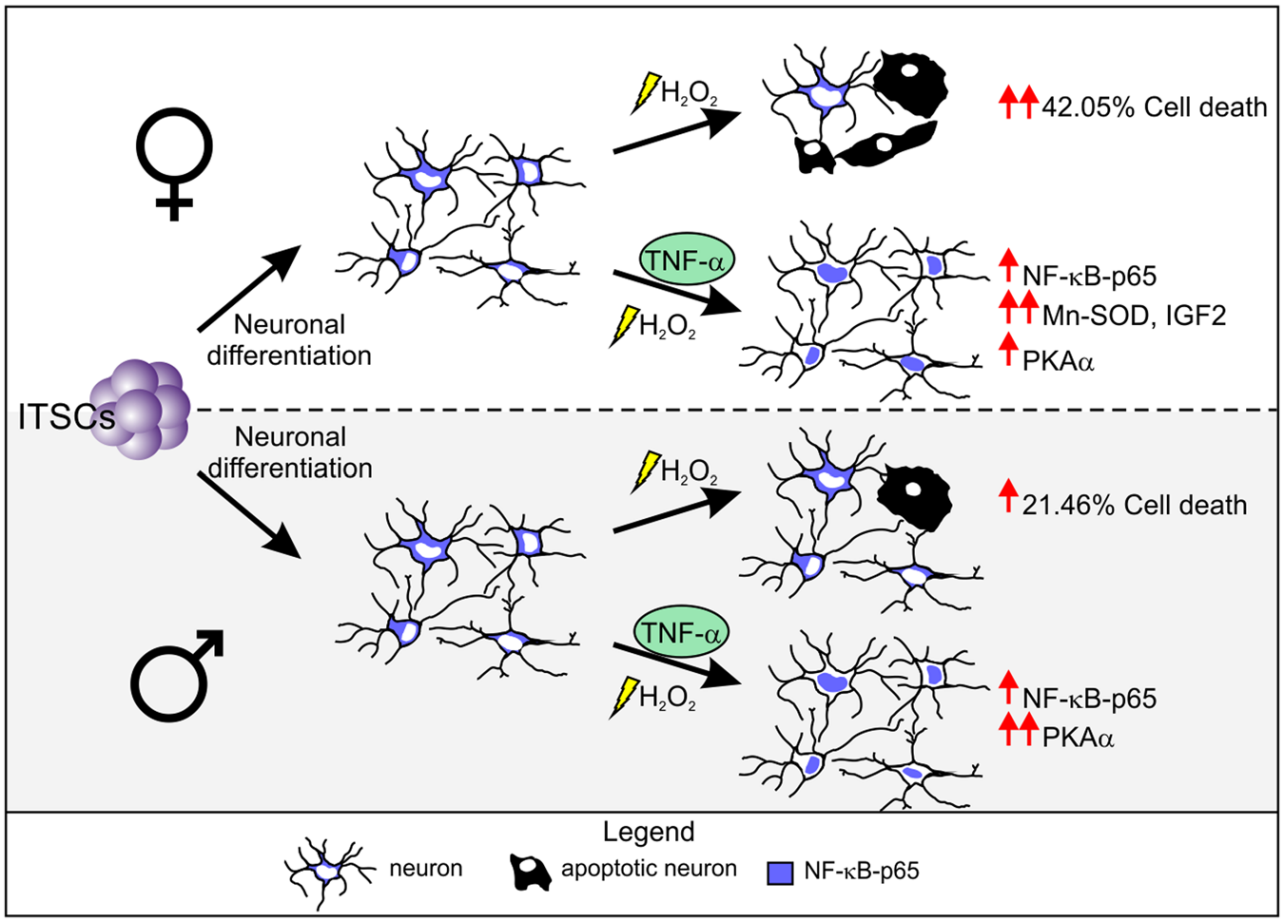
Sex-specific response to oxidative stress insult and NF-κB-mediated neuroprotection in human NCSC-derived neurons. Female ITSC-derived neurons responded with a higher sensitivity to oxidative stress-induced neuronal death, and TNF-α-mediated neuroprotection compared to their male counterparts. TNF-α-mediated neuroprotection led to an increase in NF-κB-p65 nuclear translocation, triggering differential expression of sex-specific NF-κB target genes.

In summary, we provide here evidence that NF-κB-p65 is a key player in neuroprotection of human neurons against oxidative stress in a sex-dependent manner. We demonstrate a sex-dependent difference of stress response and TNF-α-mediated neuroprotection, with a strong increase of both H_2_O_2_-mediated cell death as well as neuroprotection against cell death in female derived neurons compared to their male counterparts (Fig. 7). These differences were emphasized by the sex-specific differential expression of NF-κB-p65 target genes SOD2 and IGF2 in TNF-α-dependent neuroprotection upon oxidative stress-insult. In line with our findings, increasing evidences pointing towards sex-specific differences in risk and severity of neurodegenerative diseases, such as Alzheimer’s disease. Since oxidative stress is directly associated to neurodegenerative diseases, but little is known about the underlying molecular mechanisms of neuroprotection, NF-κB-signalling may be a crucial parameter for treatment strategies and neuronal regeneration therapies.

## Methods

All methods were performed in accordance to the relevant guidelines and regulations.

### Isolation and Cultivation of ITSCs

ITSCs were isolated from adult human inferior turbinate tissue obtained by biopsy during routine surgery after informed consent according to local and international guidelines and cultivated as described previously^36^. The ethics board of the medical faculty of the University of Münster approved all the procedures described in this article (No. 2012–015-f-S). All experiments and methods were performed in accordance to the relevant guidelines and regulations. ITSCs were cultivated within the 3D blood plasma (BP) matrix^37^, and dissociated ITSCs were resuspended in Dulbecco’s modified Eagle’s medium/Ham F-12 (DMEM/F-12; Biochrom, Berlin, Germany, http://www.biochrom.de) supplemented with basic fibroblast growth factor-2 (FGF2; 40 ng/ml; Miltenyi Biotec), epidermal growth factor (EGF; 20 ng/ml; Miltenyi Biotec) and B27 supplement (Gibco) followed by supplementation with 10% of clinically accredited therapeutic human blood plasma (BP; obtained from Institut für Laboratoriums und Transfusionsmedizin, Bad Oeynhausen, Germany) and cultivated at 37 °C, 5% O_2_ and 5% CO_2_.

### Neuronal differentiation

For neuronal differentiation, cells of six donors were expanded within the 3D BP matrix, were dissociated and resuspended in DMEM high glucose (Sigma-Aldrich) containing 200 mM L-glutamine (Sigma-Aldrich) and 10% foetal calf serum (Sigma-Aldrich) an plated at a density of 5 × 10^4^ cells per 24 well plate followed by cultivation at 37 °C, 5% CO_2_ and atmospheric O_2_ for 2 days. Afterwards, 1 μM dexamethasone (Sigma-Aldrich), 2 μM insulin (Sigma-Aldrich), 500 μM 3-isobutyl-1-methylxanthine (Sigma-Aldrich), 200 μM indomethacin (Sigma-Aldrich) and 200 μM ethanol were added to the medium to induce neuronal differentiation (neuronal induction medium, NIM). After 9 days of differentiation cells were induced adding 0.5 μM retinoic acid (Sigma Aldrich) and 1x N-2 supplement (Gibco, Darmstadt, Germany, http://www.invitrogen.com). Subsequently, the medium was changed by removing half of the volume, followed by addition of fresh pre-warmed NIM containing 1x N-2 supplement^38^. ITSCs were differentiated for 1 month, and further stimulated using different drugs, or treated for immunocytochemical and RT-PCR analysis.

### Neuronal stimulation

After 30 days of differentiation neurons were exposed to the excitatory neurotransmitter glutamate (GLU) or its agonist α-amino-3-hydroxyl-5-methyl-4-isoxazole-propionate (AMPA), the cytokine Tumour Necrosis Factor α (TNF-α, Calbiochem®), hydrogen peroxide (H_2_O_2_), and the NF-κB inhibitor pyrrolidine dithiocarbamate (PDTC)^23^. Before treatment with glutamate or AMPA, cells were washed three times with buffered control salt solution (CSS)^74^ containing 120 mM NaCl, 5.4 mM KCl, 1.8 mM CaCl2, 25 mM Tris HCl (pH 7.4), 15 mM D-glucose. For inhibitor controls cells were pre-incubated with either 1 μM dibenzocyclohepteneimine (MK-801, Tochris Bioscience, UK)^75^ or 50 μM 6-cyano-7-nitroquinoxaline-2,3-dione (CQNX, Tochris Bioscience, UK)^12^ for 10 min at 37 °C, before 10 min treatment with glutamate or AMPA respectively. After treatment with different concentrations, cells were washed with CSS and incubated with complete medium for 45 min at 37 °C. Control cells received identical incubation times and washing steps with CSS^12^. The pulse with 10 ng/ml TNF-α was performed for 30 min, 1 h, and 24 hours. For oxidative stress induction, 300 μM H_2_O_2_ were applied during 25 h and to analyse the neuroprotective role of NF-κB during oxidative stress, a pre-treatment with 10 ng/ml TNF-α was performed for 2 hours previous to the treatment with H_2_O_2_. Untreated control cells received identical incubation times. In order to confirm NF-κB activation due to TNF-α, a pre-treatment using 100 μM PDTC for one hour was performed and samples were directly used or further treated with TNF-α for at least one hour. For Indirect immunofluorescence assay cells were fixed as described below. For Smart-seq. 2, cells were directly used after treatment, whose duration was 3 h for PDTC and TNF-α treatment as well as for the combination treatments.

### Immunocytochemistry

Differentiated ITSCs were fixed for 15 min in phosphate-buffered 4% paraformaldehyde (PFA 4% pH 7.4) at room temperature (RT) followed by 3 wash steps in phosphate-buffered saline (1xPBS). The cells were permeabilized with 0.02% Triton X-100 and blocked using 5% of appropriate serum or 3% BSA for 30 minutes at RT, followed by incubation with primary antibodies for 1 hour at RT. Antibodies used were anti-neurofilament NF200 (1:200, N4142, Sigma-Aldrich), anti-MAP-2 (1:100, Sc-20172, Santa Cruz Biotechnology), anti-Synaptophysin (1:250, MAB5258, Merck Millipore), anti-vGlut2 (1:200, MAB5504, Millipore), anti-nestin (1:200, MAB5326, Millipore) anti-olig-2 (1:250, Q13516, R&D Systems), anti-NF-kappa B p65 (D14E12, Cell Signaling). The secondary fluorochrome-conjugated antibodies (1:300; goat anti-mouse Alexa 555, goat anti-rabbit Alexa 555, donkey anti goat Alexa 555; Life Technologies) were incubated for 1 hour at RT. Nuclear counterstaining was performed with 49,6-diamidino-2-phenylindole (DAPI; 1 μg/ml; Sigma-Aldrich) for 15 min at RT. Fluorescence imaging was performed using a confocal laser scanning microscope (LSM 780; Carl Zeiss, Jena, Germany) and analyzed using ZEN software from the same provider or ImageJ^76^.

### Reverse transcription Polymerase Chain Reaction

Total RNA was isolated using the TRI Reagent (Sigma-Aldrich) according to the manufacture’s guidelines. Quality and concentration of RNA were assessed via Nanodrop ultraviolet spectrophotometry. cDNAs were synthesized by reverse transcription using the First Strand cDNA Synthesis Kit (Fermentas Life Sciences). PCR was performed using the GoTaq (Promega) according to the manufacturer’s guidelines and 10 µM primers (Sigma-Aldrich). The cycling conditions comprised an initial denaturation of 1 min at 94 °C and 35–38 cycles of 15 s at 94 °C, 15 s at 60 °C, and 20 s at 72 °C followed by a final elongation for 1 min at 72 °C. For primer sequences see Table 1.

**Table 1:** Primers sequences for reverse transcription polymerase chain reaction.

Target	Sequence 5′-3′
Nestin	CAGCGTTGGAACAGAGGTTG
Rev-Nestin	GCTGGCACAGGTGTCTCAAG
MAP-2	GAGGATGAAGAGGGTGCCTT
Rev-MAP-2	AGCTCTCCGTTGATCCCATTC
Synaptophysin	TGTAGTCTGGTCAGTGAAGCC
Rev- Synaptophysin	GCAGGGCTCAGACAGATAA
AMPA receptor subunit 1	GGGCGATAATTCAAGTGTTCA
Rev-AMPA receptor subunit 1	GGCTCCGTATTTTCCATCAC
NMDA Receptor subunit 1	GCTCCTCGAGAAGGAGAACA
Rev- NMDA Receptor subunit 1	GCCATTGTAGATGCCCACTT
Vesicular glutamate transporter 1	CACAAGACTCGGGAGGAGTG
Rev- Vesicular glutamate transporter 1	GCCTCATCCTCCATTTCGCT
Glutamate metabotropic receptor 1	AGCTGCTGATTTCTCAGCCAA
Rev- Glutamate metabotropic receptor 1	GCCTCCAACATTGGAATGGA
Tyrosine Hydroxylase	CCGTGCTAAACCTGCTCTTC
Rev- Tyrosine Hydroxylase	CGCACGAAGTACTCCAGGT
Choline Transporter	GGCACAGCTGAAGCAGTTTA
Rev- Choline Transporter	CCCATGCGTTTTCCATAGAT
Serotonin transporter	CTCCGAGGACAACATCACCT
Rev- Serotonin transporter	CAGAGGTCTTGACGCCTTTC
RPLP0 (Ribosomal Protein Lateral Stalk Subunit P0)	TGGGCAAGAACACCATGATG
Rev-RPLP0	AGTTTCTCCAGAGCTGGGTTGT

### SMART-Seq2

For full-length cDNA generation, the protocol recently described by Picelli *et al*.^77^ slightly modified was applied. Approximately 20000 cells/treatment ITSC-derived glutamatergic neurons were used. Cells were harvested by centrifugation (5000 g for 5 min at RT) and directly lysed with an adjusted amount of lysis buffer (RNase inhibitor, 0.2% Triton X-100). Afterwards the annealing mix containing AccuStart Taq Polymerase HiFi (Quanta bio), oligo-dT primer, dNTP-mix, was added to the cell lysate. Probes were incubated 3 min at 72 °C, and the reverse transcription-mix containing SuperScript II reverse transcriptase (Thermo Fisher Scientific) was added. Reverse transcription, relying on template-switching reaction was performed. The cycling program comprised an initial denaturation of 90 min at 42 °C, following by 9 cycles of 2 min at 50 °C and 2 min at 42 °C followed by a final elongation for 15 min at 70 °C. The PCR pre-amplification mix was added to the first-strand reaction. PCR pre-amplification-cycling-program comprised an initial denaturation of 3 min at 98 °C and 21 cycles of 20 s at 98 °C, 15 s at 67 °C, and 6 min at 72 °C followed by a final elongation for 5 min at 72 °C.

### Real-time PCR

All Quantitative polymerase chain reactions (qPCR) were performed in triplicate using Platinum SYBR Green qPCR Super-Mix UDG (Invitrogen), according to the manufacturer’s guidelines, and assayed with a Rotor Gene 6000 (Qiagen). Primers used are listed in Supplementary Table S1.

### Hippocampal Slice Culture and transplantation of ITSCs on organotypic hippocampal slice cultures and Immunocytochemical analyzes

For organotypic hippocampal slice culture, hippocampi of mice (postnatal day 5) were isolated and rapidly cut perpendicularly to the longitudinal axis into 400 µm thick slices with a McIllwain Tissue Chopper. Slices were cultivated on culture plate inserts according to De Simoni and Yu^78^. In parallel ITSCs were transduced with lentivirus pFUGW containing a constitutively expressed GFP-gene under control of human ubiquitin c promoter. GFP^+^-ITSCs and ITSCs were cultivated as neurospheres for 2 days. 7 days after slice preparation, dissociated cells (1 × 10^4^) were dropped onto each slice following by cultivation for 14 days at 37 °C and 5% CO_2_. Slices were cut off from the membrane and free-floating fixated in PFA 4% for 1 h at 4 °C on agitation. After 3 washes with PBS, slices were incubated in PBS containing 0.1% Triton X-100 and 5% goat serum for 1 hour at RT. Slices containing transplanted GFP^+^-ITSCs were double immuno-labeled with anti-GFP (1:1000, sc-9996, Santa Cruz) combined with anti-synaptophysin (1:200) or anti-vGlut2 (1:200). Slices containing transplanted non transduced ITSCs were free-floating stained with anti-human nuclei (HuNu, 1:200, MAB1281, Millipore) combined with anti-MAP-2 (1:100), or anti-Gat1 (1:200, AB1570, Millipore) for 48 h at 4 °C. Respective secondary fluorochrome-conjugated antibodies (goat anti-mouse Alexa 555, goat anti-rabbit Alexa 555, donkey anti-rabbit Alexa 488, donkey anti-mouse Alexa 488) were applied for 2 h at RT under exclusion of light. Nuclei were stained with TOTO®-3 Iodide (642⁄660 nm, life technologies).

### Cell Counting and Statistics

Quantification of immunofluorescence staining was performed for minimum 3 different donors. For each time point 6–12 pictures were analysed per donor, where the mean of the nuclear integrated density was measured by defining the region of interest with the nuclear DNA channel using ImageJ^76^. For analysis of neuronal survival the same channel was used to analyse the nuclear chromatin morphology. Nonviable neurons were recognized by nuclear condensation and/or fragmented chromatin. In phase contrast images, those neurons were irregularly shaped with shrunken cell body and/or disrupted neurites. The number of viable and nonviable neurons was counted in four to five field pictures and death rate was calculated. Data was further analysed for statistics using Past3^79^ and/or GraphPad Prism 5 (GraphPad software, La Jolla, CA, http://www.graphpad.com). Normality of the data sets was refuted after analysis using Kolmogorov-Smirnov and Shapiro-Wilk normality tests. Non-parametric Kruskal-Wallis test was used to compare the medians between the different data sets for the different donors (***p ≤ 0.001). Non-parametric Mann-Whitney test was used to compare two pair of groups (***p ≤ 0.001). Further analysis was performed using Tukey’s test (*p ≤ 0.05, **p ≤ 0.01, ***p ≤ 0.001).

## Electronic supplementary material

Supplementary information
