# Supplementary material for: NF-κB p65 directs sex-specific neuroprotection in human neurons
Source: Sci Rep. 2018 Oct 30;8:16012. doi: 10.1038/s41598-018-34394-8 (PMC6207661; doi:10.1038/s41598-018-34394-8)
Supplement: Supplementary file 1 — Supplementary information [file 41598_2018_34394_MOESM1_ESM.pdf]

Supplementary information

NF-κB p65 directs sex-specific neuroprotection in human neurons

Lucia M Ruiz-Perera[1], Lennart Schneider[1], Beatrice A Windmöller[1], Janine Müller[1], Johannes FW Greiner[2], Christian Kaltschmidt[2] and Barbara Kaltschmidt[1,2*]

Supplementary Table S1: Primer sequences for real-time polymerase chain reaction.

Target	Sequence 5'-3'
IGF1 (Insulin-like growth factor 1)	GAAGATGCACACCATGTCCT
Rev- IGF1	TAAAAGCCCCTGTCTCCACA
IGF2 (Insulin-like growth factor 2)	TGGACTTGAGTCCCTGAACC
Rev- IGF2	GATGGCCAGTTTACCCTGAA
PKAcatα (c-AMP-dependent protein kinase catalytic subunit α)	TACAACAAGGCCGTGGACTG
Rev- PKAcatα	AGATCTGGATGGGCTGGTCT
SOD2 (Superoxide dismutase 2)	TGGAAGCCATCAAACGTGACT
Rev- SOD2	TTGAAACCAAGCCAACCCCA
cIAP1 (Cellular inhibitor of apoptosis protein 1)	CCACTGGAGAAGAAAATGCTG
Rev- c-IAP1	CCAAGGCAGATTTAACCACA
c-IAP2 (Cellular inhibitor of apoptosis protein 2)	GACAGCCCAGGAGATGAA AA
Rev- c-IAP2	CACGGCAGCATTAATCACAG
RPLP0 (Ribosomal Protein Lateral Stalk Subunit P0)	TGGGCAAGAACACCATGATG
Rev-RPLP0	AGTTTCTCCAGAGCTGGGTTGT
β-actin (beta actin)	TCCCTGGAGAAGAGCTACGA
Rev- β-actin	AGCACTGTGTTGGCGTACAG

Supplementary Figure 1

Full-length figures of gels showing the RT-PCR of differentiated ITSCs. The grouped gels in Fig. 1k were cropped from different parts of the same gel or from different gels as depicted here. In particular, mRNA levels were analysed for Nestin, MAP-2, Synaptophysin, AMPA receptor subunit 1, NMDA Receptor subunit 1, vesicular glutamate transporter 1, and glutamate metabotropic receptor 1 after neuronal differentiation. RPLP0, GAPDH and eEF2 served as housekeeping genes. NTC: non-template-control. The first 4 figures show gels ran in parallel on the same experiment, the last two gels also ran in parallel. For the last gels, two donors were shown after 0 and 30 days of differentiation respectively.

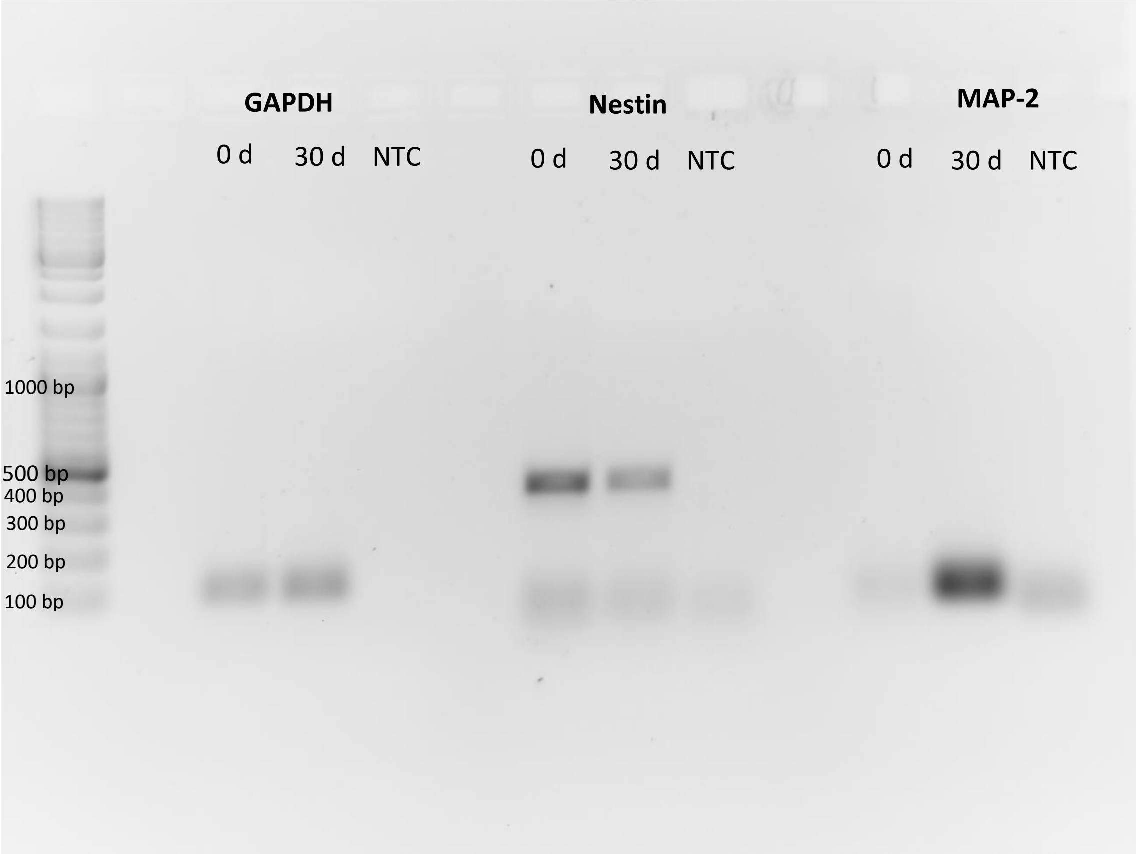

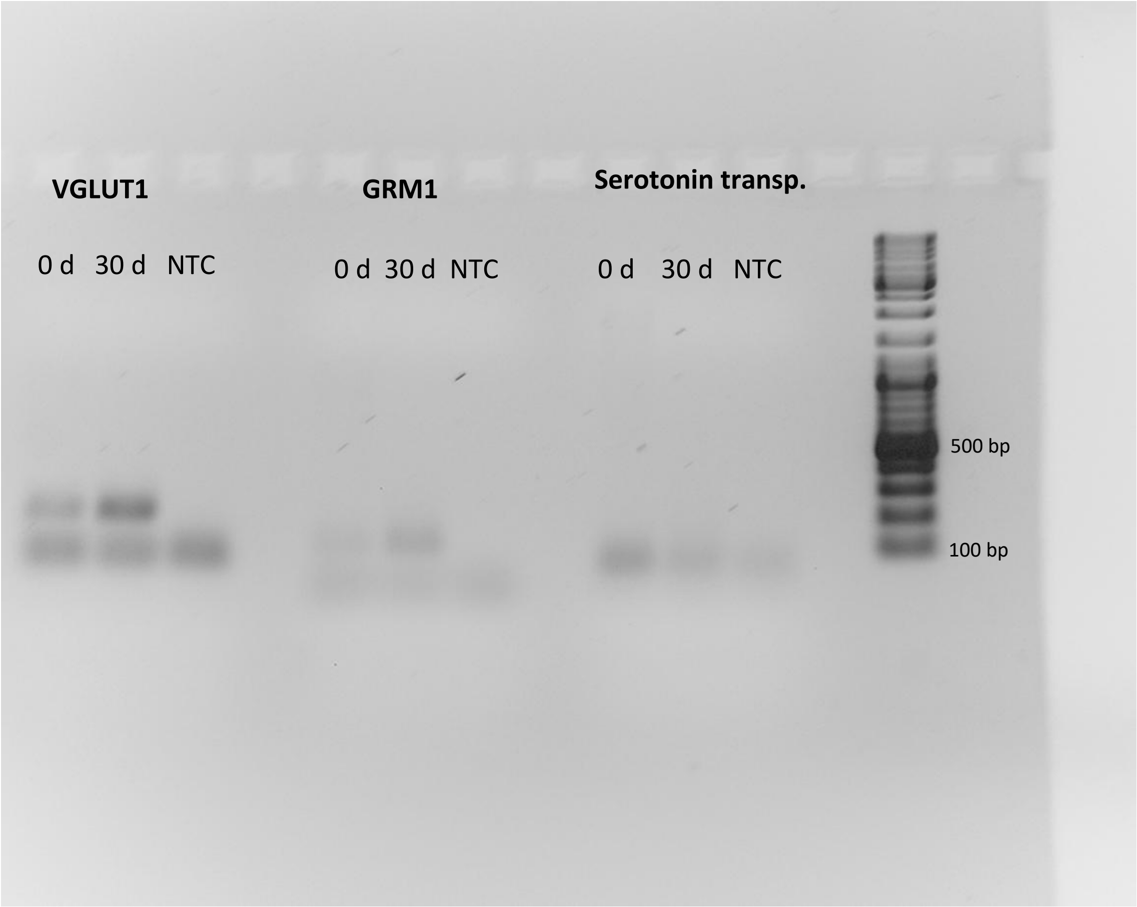
VGLUT1
GRM1
Serotonin transp.
0 d 30 d NTC
0 d 30 d NTC
0 d 30 d NTC
500 bp
100 bp

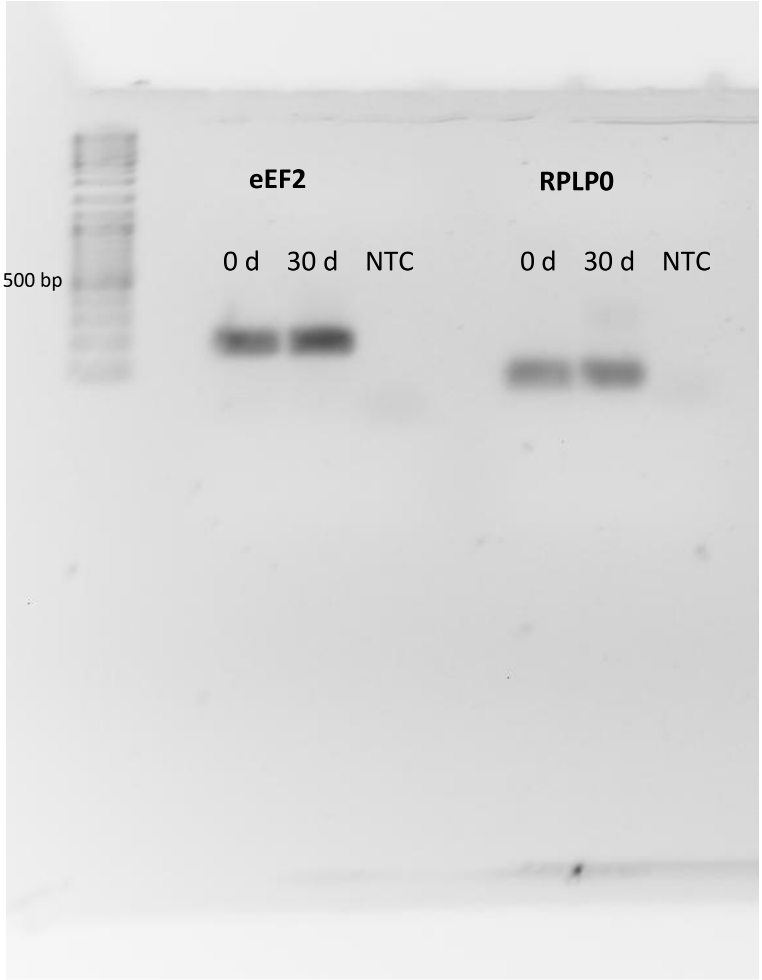
eEF2
RPLP0
0 d
30 d
NTC
0 d
30 d
NTC
500 bp

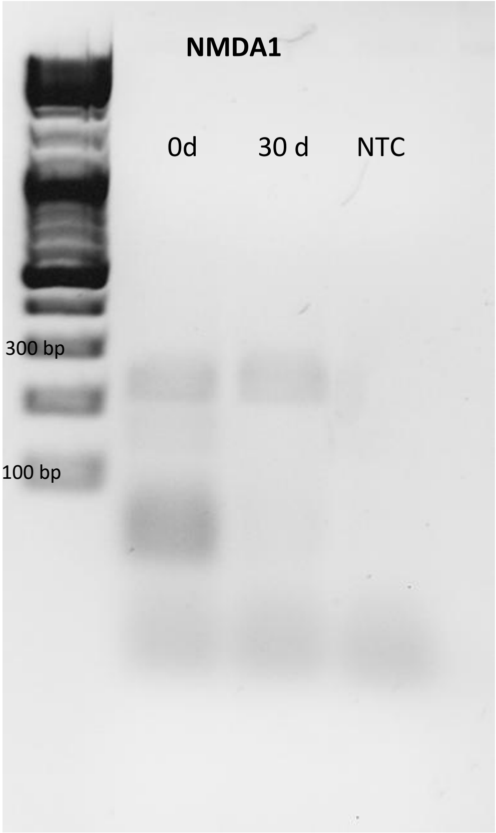
NMDA1
0d
30 d
NTC
300 bp
100 bp

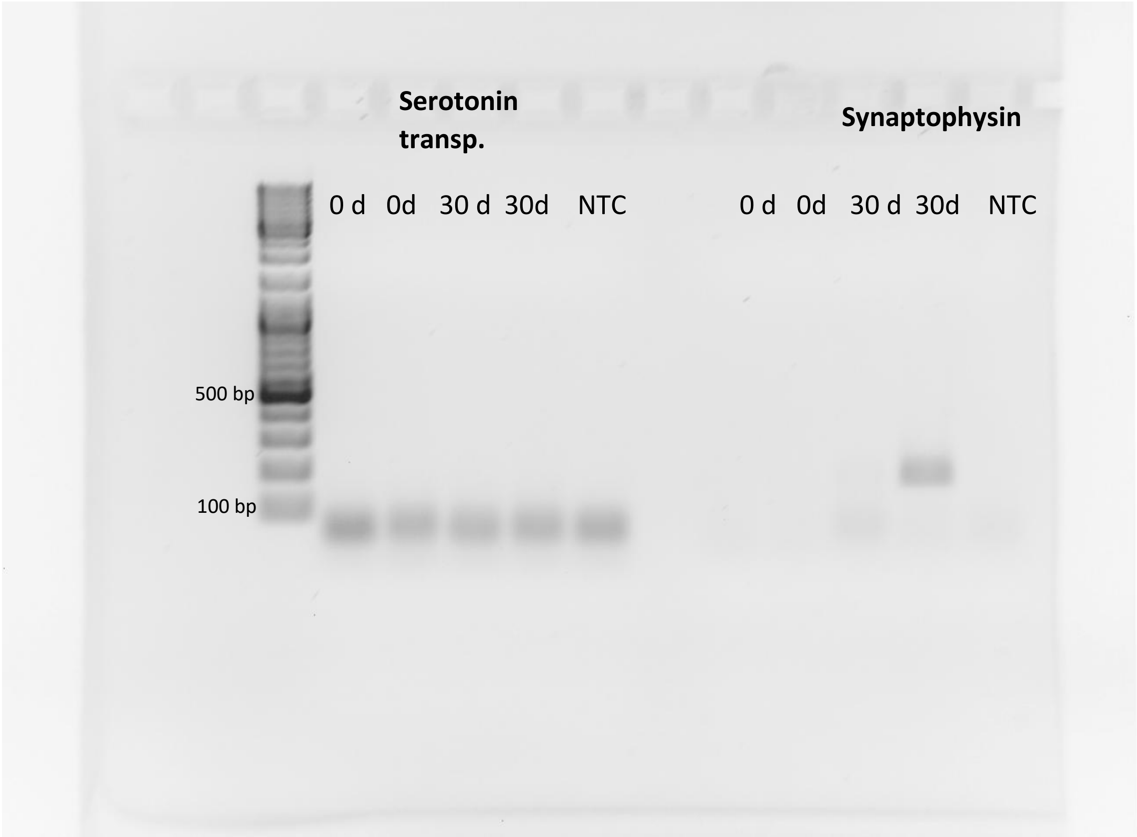
Serotonin
transp.
Synaptophysin
0 d
0d
30 d
30d
NTC
0 d
0d
30 d
30d
NTC
500 bp
100 bp

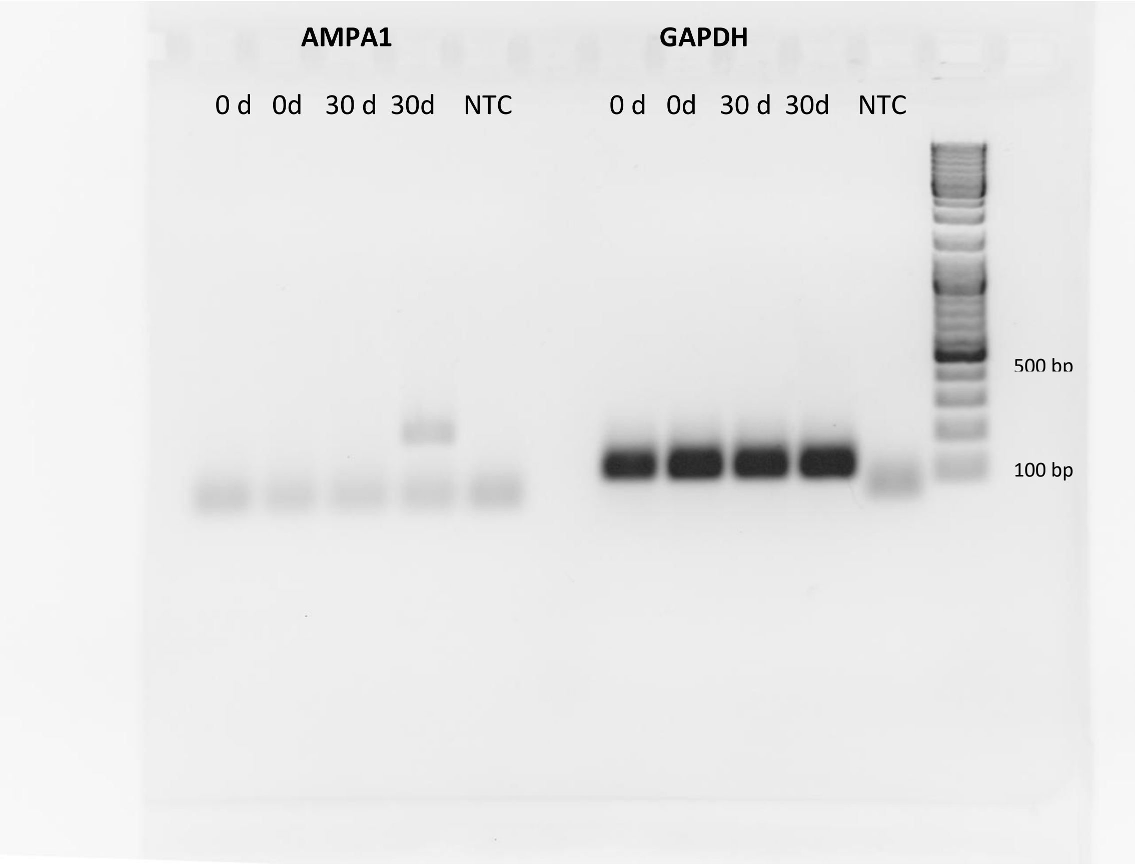
AMPA1
GAPDH
0 d 0d 30 d 30d NTC
0 d 0d 30 d 30d NTC
500 bp
100 bp
